# Analysis of Umbilical Artery Hemodynamics in Development of Intrauterine Growth Restriction Using Computational Fluid Dynamics with Doppler Ultrasound

**DOI:** 10.3390/bioengineering11111169

**Published:** 2024-11-20

**Authors:** Xue Song, Jingying Wang, Ke Sun, Chunhian Lee

**Affiliations:** 1 School of Energy and Power Engineering, Shandong University, Ji’nan 250061, China; 202120595@mail.sdu.edu.cn (X.S.); sunkeke@sdu.edu.cn (K.S.); leechx@sdu.edu.cn (C.L.); 2 School of Aeronautic Science and Engineering, Beihang University (BUAA), Beijing 100191, China

**Keywords:** intrauterine growth restriction (IUGR), umbilical artery (UA), blood flow, Doppler ultrasound, computational fluid dynamics (CFD)

## Abstract

Intrauterine growth restriction (IUGR), the failure of the fetus to achieve his/her growth potential, is a common and complex problem in pregnancy. Clinically, IUGR is usually monitored using Doppler ultrasound of the umbilical artery (UA). The Doppler waveform is generally divided into three typical patterns in IUGR development, from normal blood flow (Normal), to the loss of end diastolic blood flow (LDBF), and even to the reversal of end diastolic blood flow (RDBF). Unfortunately, Doppler ultrasound hardly provides complete UA hemodynamics in detail, while the present in silico computational fluid dynamics (CFD) can provide this with the necessary ultrasound information. In this paper, CFD is employed to simulate the periodic UA blood flow for three typical states of IUGR, which shows comprehensive information on blood flow velocity, pressure, and wall shear stress (WSS). A new finding is the “hysteresis effect” between the UA blood flow velocity and pressure drop in which the former always changes after the latter by 0.1–0.2 times a cardiac cycle due to the unsteady flow. The degree of hysteresis is a promising indicator characterizing the evolution of IUGR. CFD successfully shows the hemodynamic details in different development situations of IUGR, and undoubtedly, its results would also help clinicians to further understand the relationship between the UA blood flow status and fetal growth restriction.

## 1. Introduction

Intrauterine growth restriction (IUGR), also known as fetal growth restriction, is defined as fetuses with an estimated fetal weight or abdominal circumference below the 10th percentile for gestational age [1]. IUGR occurs in approximately 8% to 10% of pregnancies [2]. Fetal growth restriction is associated with a variety of adverse perinatal outcomes. The risk of intrauterine demise in IUGR fetuses increases with decreasing fetal weight [3]. Newborns with growth restriction are susceptible to complications, such as hypoglycemia, hyperbilirubinemia, intraventricular hemorrhage, respiratory distress syndrome, and neonatal death [4,5,6,7,8]. Some studies have revealed that these fetuses are predisposed to lower childhood cognitive outcomes and diseases such as obesity, type 2 diabetes mellitus, coronary artery disease, and stroke in adulthood [9,10].

**Figure 1:**
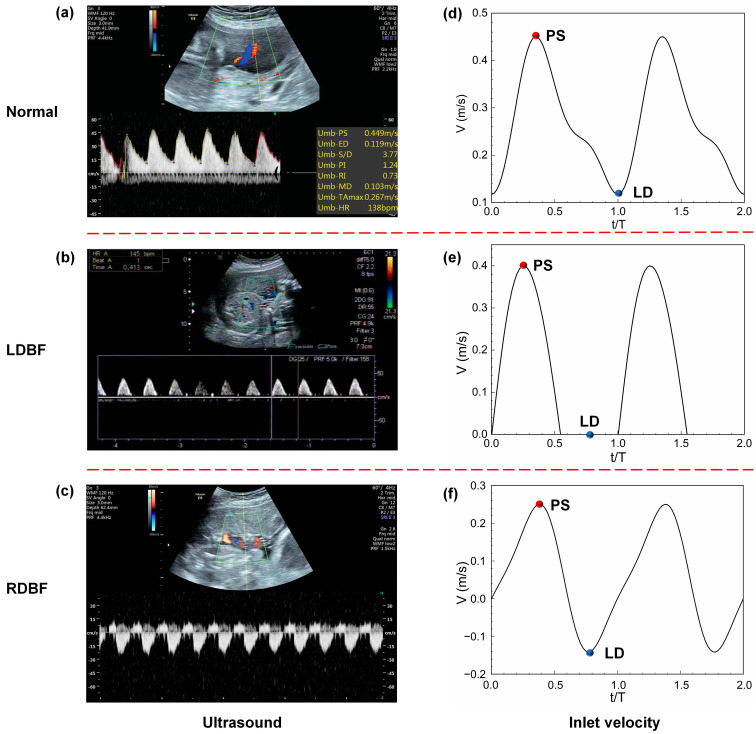
The UA color and pulsed Doppler ultrasound spectrogram (**a**–**c**) and inlet velocity profile (**d**–**f**) for Normal (**a**,**d**), LDBF (**b**,**e**), and RDBF (**c**,**f**). The “PS” and “LD” moments represent the peak systolic and least diastolic, respectively. The flow time (*t*) is normalized by the time of one cardiac cycle (T). In Doppler ultrasound images, blue indicates blood flow traveling away from the transducer (negative Doppler shift), and red indicates blood flow traveling toward the transducer (positive Doppler shift).

The monitoring of growth-restricted fetuses is mainly dependent on umbilical artery (UA) time–velocity waveforms obtained by pulsed Doppler ultrasound [11]. Based on the severity of the IUGR, the ultrasound velocity waveform is derived from a normal periodic signal (Normal) that may gradually develop into a lost diastolic signal (LDBF) or even a diastolic signal with reflux (RDBF), as shown in Figure 1a–c. Current ultrasound technology hardly shows the details of blood flow, such as the flow structure, pressure distribution, and wall shear stress (WSS), which can help to understand the disease better and improve treatment.

Recently, many studies of human hemodynamics have been successfully performed using in silico computational fluid dynamics (CFD), a method of solving the governing equations of blood flow on the computer [12,13,14,15,16]. It has the advantages of free settings, controlled conditions, group scalability, low time–economic costs, and noninvasiveness [17,18,19]. There are two main types of CFD applications for hemodynamic studies of the cardiovascular system. On the one hand, there is the evaluation of treatments before and after implanting medical devices in patients, such as heart valves, stents, and filters [10,20,21,22,23]. On the other hand, there is the simulation of hemodynamic changes in the vasculature induced by diseases. For example, vascular morphing due to hemangiomas, enlargement, hyperplasia, stenosis [24,25,26,27,28,29,30], and changes in intravascular hemodynamics due to abnormal blood flow conditions [31,32,33]. There have been studies using CFD for hemodynamic analysis within the fetal UA to reveal blood flow patterns, including blood flow patterns specific to normal and abnormal geometries, and the heat exchange between the placenta and the fetus has also been calculated [34,35,36]. CFD methods are also involved in the disease research of IUGR. Saw et al. [37] characterized the WSS of 22 normal and 21 IUGR pregnancies, unifying all states in the IUGR evolutionary course into one single state for the study.

Currently, the studies on the hemodynamic properties associated with IUGR are still scarce. Clinically, fetal blood flow parameters can mainly be read from pulsed Doppler ultrasound. The previous CFD studies of IUGR flow do not take into account all three states of IUGR with the unsteady pulsatility of the UA flow condition. This work considers the cyclically pulsatile nature of UA blood flow and systematically analyzes hemodynamic characteristics in all three typical development states of IUGR.

## 2. Materials and Methods

### 2.1. Subjects

Pregnant subjects were recruited in a retrospective study between January 2019 and December 2021 in the Department of Obstetrics and Gynecology, Jinan Central Hospital. Each subject gave their informed consent in accordance with the Declaration of Helsinki. Clinical data were collected from the medical anamnesis; ultrasound parameters were taken between the 24th and 28th weeks of gestation. Subjects were diagnosed with IUGR if the estimated fetal weight in the 20th week of gestation was below the 10th percentile. Patients with twin pregnancies, preterm births, genetic malformations, chromosomal or developmental disorders, hypertension (<140/80 mmHg), diabetes (fasting plasma glucose < 6.9 mmol/L measured in the 24th week of gestation), or alcohol, nicotine, or drug abuse were excluded from this study. The patients involved in this study did not have immunological, cardiovascular, gastrointestinal, or pulmonary disease.

### 2.2. Ultrasound Image Acquisition

Every ultrasound examination was performed by the same person to avoid interobserver variability. All patients were scanned in a semi-recumbent position. The factorial default setting “Obstetrics/2–3 trimester” was used in 2D mode on a GE E8 or TOSHIBA 500 machine. Gestational age was determined based on the first day of the last menstrual period and on ultrasound biometry (crown–rump length and biparietal diameter) between the 9th and 11th weeks of pregnancy. In the 20th–24th weeks, fetal biometry was used to assess the estimated fetal weight by the formula B of Hadlock [38].

A conventional color Doppler study of the umbilical arteries was performed, and the parameters of S/D ratio, PI, and RI were read from the ultrasound report. The fetal cardiac cycle, T, was uniformly 0.4 s. The UA ultrasound spectrogram is shown in Figure 1a–c.

### 2.3. UA Model Reconstruction

In anatomy, the longitudinal section of the umbilical cord (UC) shows two UAs and an umbilical vein (UV) in a spiral arrangement [39]. Previous studies have treated the UA as a spiral tube in geometry [33,36,37], and this study follows this pattern. The main parameters of the UA include the helical radius, cord length, and pitch. The standard parameters of the UA model in the present study are taken from the UC Doppler ultrasound data in fetuses with IUGR measured by Raio et al. [40]. Assuming that the UA and UV are circular in cross-section, the radii of the UA and UV are obtained to be 3.82 mm and 5.5 mm, respectively. The spiral radius is calculated as follows:

(1) $$R_{r}=R_{A}+R_{V}$$

where *R_r_* is the spiral radius of the UA; *R_A_* is the radius of the UA; and *R_V_* is the radius of the UV.

The umbilical coiling index (*UCI*) and UC length are important clinical reference data, with a mean *UCI* of 0.21 ± 0.07 [39]. The *UCI* is defined as follows [40]:

(2) $$UCI=\frac{U}{L}$$

where *U* is the number of UA spirals, and *L* is the length of the UC (units: cm). The *UCI* is 0.2, and the length of the umbilical cord is taken as 30 cm to exclude the effect of abnormal *UCI* and abnormal cord length on blood flow. The UA pitch (*P_h_*) is 50 mm and is calculated as follows:

(3) $$P_{h}=\frac{L}{U}$$

**Figure 2:**
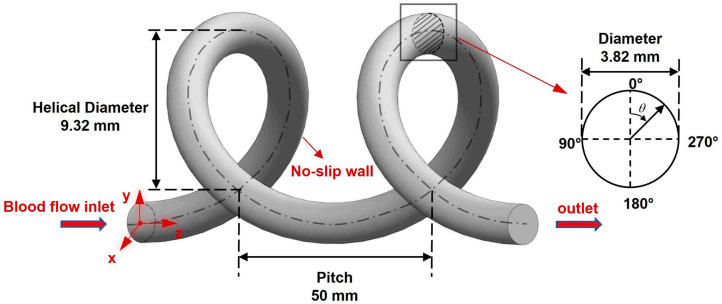
The UA model, showing only two spirals.

Based on the above parameters, the 3D reconstruction is completed by feature sweeping after drawing the baseline in SolidWorks 2022 software (Dassault Systèmes, Corp., Suresnes, France). The model is shown in Figure 2, demonstrating only two spirals with blood flow along the *z*-axis.

### 2.4. CFD Methods

#### 2.4.1. Governing Equations

The UA blood flow is governed by the viscous, incompressible Navier–Stokes equations [25,28] as follows:

(4) $$\left\{\begin{array}{l} \rho \left[\partial \vec{u}/\partial t+\left(\vec{u}\cdot \nabla \right)\vec{u}\right]=-\nabla p+\nabla \cdot \vec{\tau }\\ \nabla \cdot \vec{u}=0 \end{array}\right.$$

where $\vec{u}$ is the fluid velocity vector; *p* is the pressure; *ρ* is the blood density with a value of 1060 kg/m^3^; and $\vec{\tau }$ is the viscous stress tensor as a function of velocity [21,22]:

(5) $$\vec{\tau }=\mu \left[\nabla \vec{u}+{\left(\nabla \vec{u}\right)}^{T}\right]$$

where *μ* is the blood viscosity. Blood is a shear-thinning fluid, and its non-Newtonian property is calculated by the Carreau model as follows [22,41]:

(6) $$\mu =\mu_{\infty }+\left(\mu_{0}-\mu_{\infty }\right){\left[1+{\left(\lambda \gamma \right)}^{2}\right]}^{(n-1)/2}$$

where *γ* is the local shear rate; *λ* is the time constant with a value of 3.313 s; *n* is the power exponent with a value of 0.3568; *μ*_0_ is the zero shear viscosity with a value of 0.056 Pa·s; and *μ*_∞_ is the infinite shear viscosity with a value of 0.00345 Pa·s.

In the present study, the Reynolds number is defined as follows:

(7) Rem=ρUmDμa

where *ρ* is the density of blood, *D* is the diameter of the UA, *U_m_* is the maximum average speed of blood flow in the UA, and *μ_a_* is the average dynamic viscosity of blood. According to the following simulation data, *U_m_* and *μ_a_* are about 0.27 m/s and 0.004 Pa·s. The corresponding Re is 273, which is smaller than 2000 and means that the blood flow only needs to be considered as laminar for simulation.

#### 2.4.2. Simulation Conditions and Solver

Solving Equation (4) still requires boundary and initial conditions. The UA wall is set to be no-slip. The UA end connected to the fetus is set as the inlet of blood flow with the time-varying velocity profiles shown in Figure 1. The UA end connected to the placenta is set as the flow outlet with a pressure of 20 mmHg. The reference pressure is set at 5 mmHg to simulate the intrauterine environment. Both the outlet and reference pressures are taken from Shah et al. [33] and fall within clinically observed ranges [42,43].

The 3D double-precision solver in FLUENT 2022 R2 software (ANSYS, Inc., Canonsburg, PA, USA) is chosen for all cases. The pressure-based transient solver and the coupled mode are used in the pressure–velocity coupling method. The second-order upwind scheme is set to discretize the momentum equations. There are two requirements for achieving convergence: one is meeting the normalized continuity (with residuals below 10^−3^) and velocity convergence criteria (with residuals below 10^−5^), and the other is achieving a stable periodic variation in inlet pressure. To obtain sufficiently stable periodic results, twenty successive cycles are calculated. The date during the seventeenth cycle is used for analysis.

#### 2.4.3. Grid and Time Marching Independence Analysis

**Figure 3:**
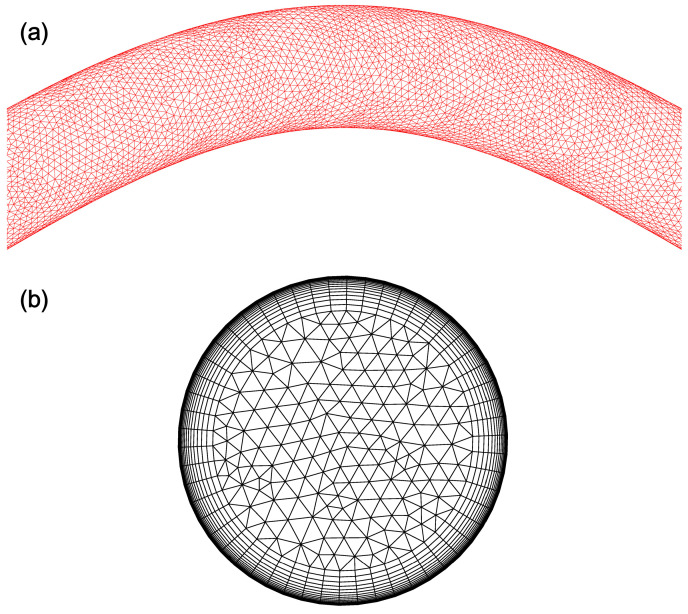
Grids with long and short cross-sections: (**a**) long cross-section; (**b**) short cross-section.

In this study, the 3D unstructured grid is created and encrypted near the UA wall. The constant inlet velocity is set as 0.6 m/s to verify the grid independence. Three sets of grids are used with 440,000, 790,000, and 1,420,000 cells. The pressure drop between the UA inlet and outlet and the area-weighted average WSS (*WSS_avg_*) at the UA wall are used as the target parameters for the grid independent study, as shown in Table 1. The *WSS_avg_* is formulated as follows [21]:

(8) $$WSS_{avg}=\frac{1}{\varphi }\int_{\varphi }WSS\cdot d\varphi$$

where *φ* is the surface area of the UA wall. From Mesh 2 to Mesh 3, the total number of grids increases by approximately 80%, while the target parameter changes are less than 0.5%. Therefore, to reduce the computational load and maintain result accuracy, Mesh 2 is selected for the calculations. Figure 3a,b show the long and short cross-sections.

**Figure 4:**
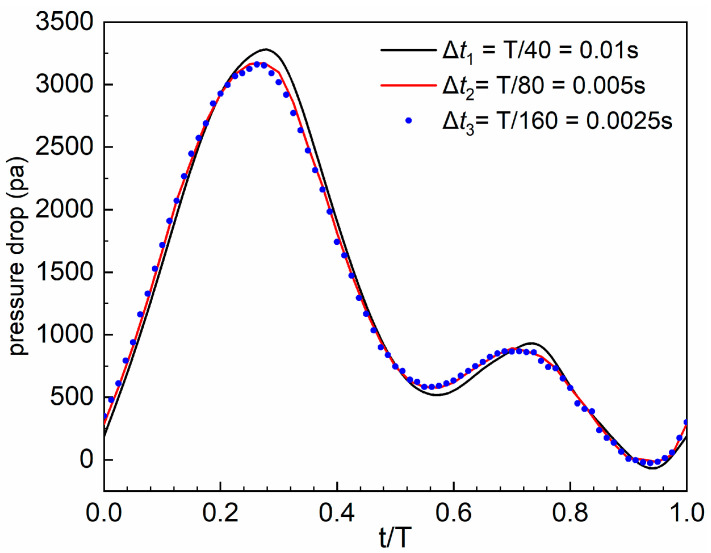
Time marching independence analysis. The flow time (*t*) is normalized by the time of one cardiac cycle (T).

After determining the suitable mesh type and size, time marching independence analysis is performed using the “Normal” case as an example. The effect of the time step on the results is evaluated by assessing the pressure drop. Three distinct time marching steps, Δ*t*_1_, Δ*t*_2_, and Δ*t*_3_, corresponding to T/40 (0.01 s), T/80 (0.005 s), and T/160 (0.0025 s), respectively, are specified. As illustrated in Figure 4, the results for Δ*t*_2_ and Δ*t*_3_ show minimal discrepancy; however, using Δ*t*_3_ as the time marching step would markedly increase the computation time. Therefore, the time marching step of T/80 (0.005 s) is adopted in this study.

#### 2.4.4. CFD Validation

**Figure 5:**
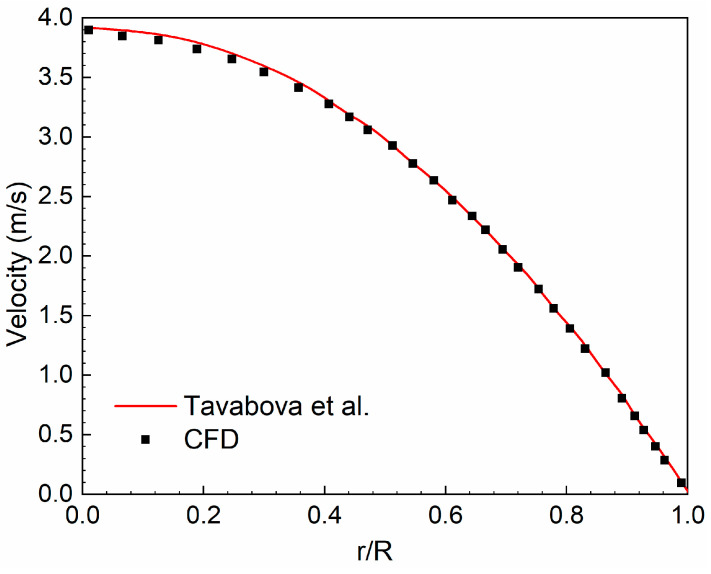
Non-Newtonian blood flow simulation validation (comparison with the theoretical solution proposed by Tevaboba et al. [46]).

FLUENT, the CFD solver in this work, has been regarded as a successful tool to simulate blood flows in many studies [21,22,37]. However, for UA blood flow, it is very difficult to take clinical measurement in vivo; it is also generally forbidden in medical ethics, and at the same time, it is very challenging to prepare a fluid with the accurate non-Newtonian property as blood for flow experiments in vitro [44,45]. Therefore, there is a lack of available clinical or experimental data to directly validate the present CFD models. Alternatively, this work uses a theoretical solution of the non-Newtonian blood flow in a tube based on the Carreau model proposed by Tabakova et al. [46] to validate the reliability of the CFD solver. In the case presented by Tabakova et al., the tube radius, *R*, is 0.0031 m, the blood flow rate is 0.0598 L/s, and the blood density is 1000 kg/m^3^. As shown in Figure 5, the CFD results under the current parameter settings agree with the test data well, indicating that the present CFD solver is reliable for blood flow simulation.

## 3. Results

The present study simulates the periodic blood flow inside the UA for all three typical development states of IUGR, Normal, LDBF, and RDBF, including the blood flow velocity, pressure, and WSS. The hemodynamic characteristics at the peak systolic (PS) and least diastolic (LD) moments labelled in Figure 1 are importantly analyzed.

### 3.1. Blood Flow Velocity

**Figure 6:**
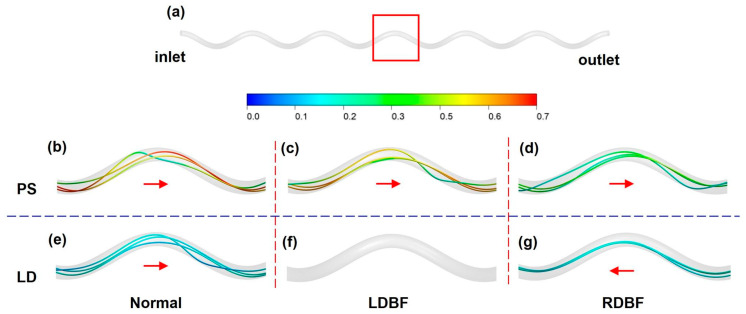
Streamlines of blood flow within the middle spiral of the UA at the peak systolic (PS (**b**–**d**)) and least diastolic (LD (**e**–**g**)) moments for Normal (**b**,**e**), LDBF (**c**,**f**), and RDBF (**d**,**g**) (in m/s): (**a**) The middle spiral of the UA is shown. The curves in the UA are the streamlines. The arrows represent the actual direction of flow.

**Figure 7:**
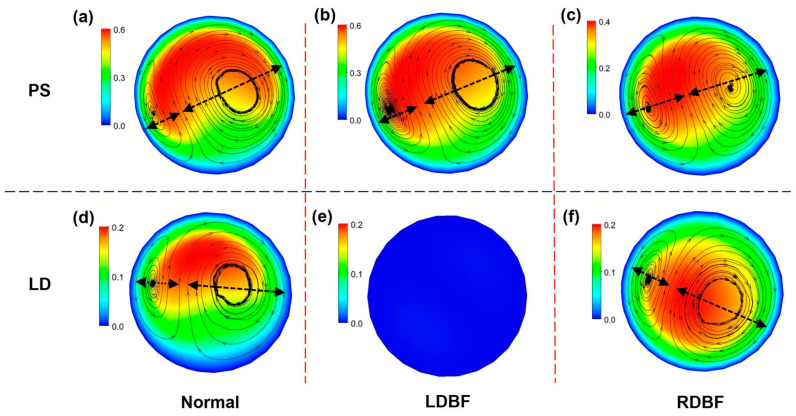
Blood flow velocity distribution in the middle cross-sections of the UA at the peak systolic (PS (**a**–**c**)) and least diastolic (LD (**d**–**f**)) moments for Normal (**a**,**d**), LDBF (**b**,**e**), and RDBF (**c**,**f**) (in m/s). The length of the arrows represents the size of the vortex diameter.

The blood flow velocity distribution of the UA at the PS and LD moments for all three typical development states is shown in Figure 6 and Figure 7. At the LD moment, there is no flow in the vessel in the LDBF state, and the flow is reversed in the vessel in the RDBF state. Affected by the spiral structure of the UA, the spiral secondary flow occurs as blood flows through the artery [47]. Due to the viscous no-slip wall boundary condition, the blood flow velocity at the UA wall is constantly zero, while the maximum flow speed occurs off-center. At systole, the regions of velocity off-center are essentially the same for all three states; at diastole, the velocity off-center for Normal and RDBF is not in the same area, while the velocity of LDBF is zero due to no flow. There is a pair of vortices of different sizes in the middle cross-section influenced by the helical secondary flow, as shown in Figure 7. The small vortices rotate counterclockwise, with the large vortices rotating clockwise, and the direction of the helix remains the same throughout one whole cycle for all three states. The diameters of the small and large vortices and the ratio of the diameters of the small and large vortices in the middle cross-sections of the UA at the PS and LD moments are shown in Table 2. The diameters of the small and large vortices of the RDBF at the PS moment are closest to each other, both being almost half of the UA diameter. Other than that, the size difference between the small and large vortices for other cases is large, with the large vortex being more than twice the diameter of the small vortex.

**Figure 8:**
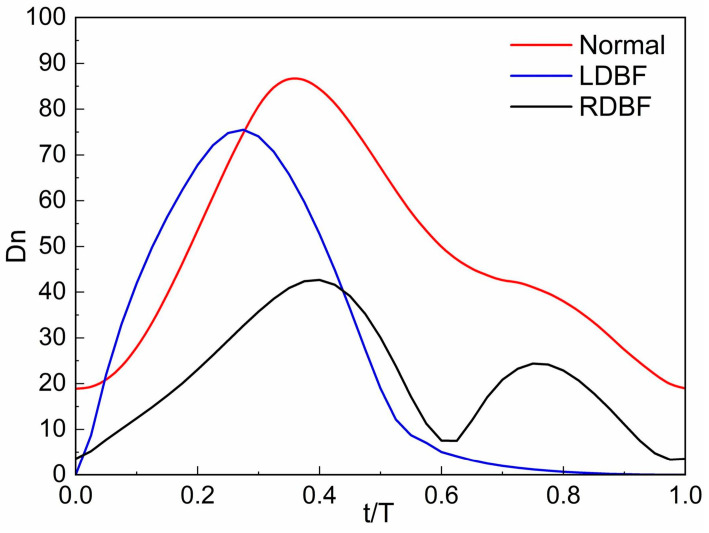
Intensity of secondary flow in the middle cross-sections of the umbilical artery for Normal, LDBF, and RDBF during a whole cycle. The flow time (*t*) is normalized by the time of one cardiac cycle (T).

It is important to measure the strength of the secondary flow. In this paper, the normalized Dean [48] is chosen to calculate the strength of the secondary flow in the spiral vessel of the UA, which is defined as follows:

(9) Dn=Rek0.5

where Re is the Reynolds number, and Re is selected for the middle cross-section away from the inlet and outlet of the UA; k is the normalized curvature, defined as follows:

(10) $$k=\frac{R_{r}\cdot R_{A}}{{R_{r}}^{2}+b^{2}}$$

where 2π*b* is the pitch. According to Equation (8), the secondary flow intensities of the UA for the three states during a whole cycle are calculated as shown in Figure 8. Overall, the intensity of the normal UA secondary flow is generally 20–40% greater than that in the RDBF and LDBF UA. However, the secondary flow intensity in the LDBF state is slightly greater than that in the Normal state from *t* = 0.05T to *t* = 0.275T. The secondary flow intensity of RDBF is greater than that of LDBF during the end systolic and entire diastolic phase.

### 3.2. Pressure

**Figure 9:**
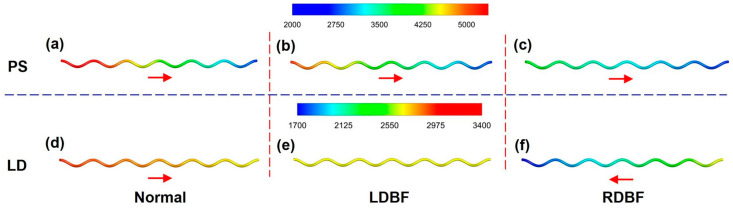
The UA pressure at the peak systolic (PS (**a**–**c**)) and least diastolic (LD (**d**–**f**)) moments for Normal (**a**,**d**), LDBF (**b**,**e**), and RDBF (**c**,**f**) (in Pa). The arrows represent the actual direction of flow.

**Figure 10:**
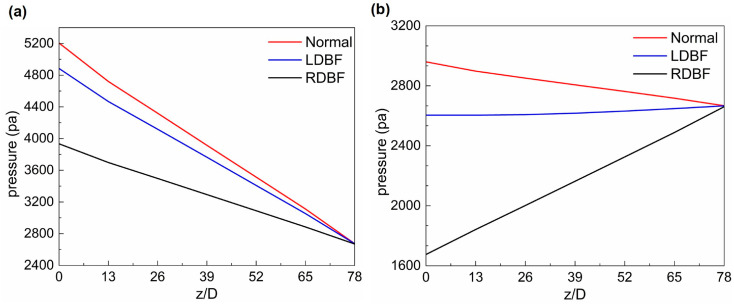
The UA pressure distribution in the flow direction normalized by the UA diameter (*D*) for Normal, LDBF, and RDBF: (**a**) the peak systolic moment; (**b**) the least diastolic moment.

For the incompressible tube laminar flow, the pressure is almost constant in the cross-section and mainly varies in the flow direction. Therefore, the pressure at the UA wall can represent the pressure of the whole UA. The wall pressure distributions of the UA under all three states are shown in Figure 9 and Figure 10. At systole, the UA pressures decrease almost linearly in the flow direction for all three cases. At diastole, the UA pressures of the Normal and RDBF states still decrease in the flow direction; it was noticed that the RDBF blood flow reverses, while the LDBF pressure changes very little due to no flow.

**Figure 11:**
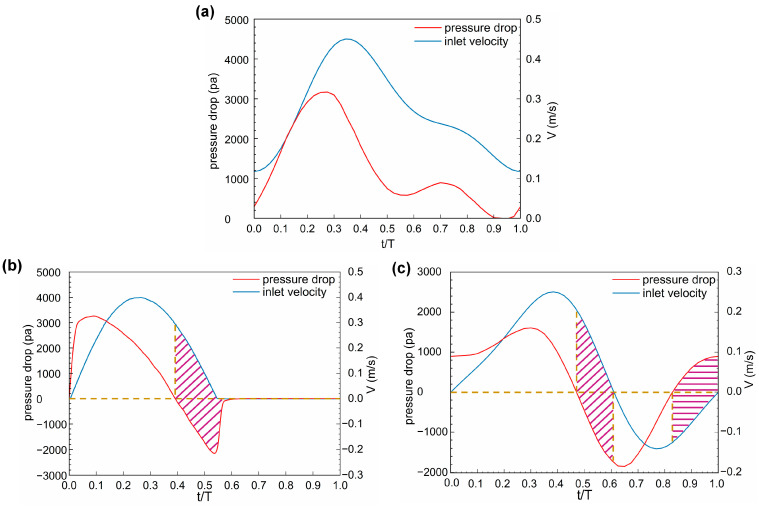
Hysteresis effect between the blood flow velocity and pressure during one cardiac cycle: (**a**) Normal; (**b**) LDBF; and (**c**) RDBF. The flow time (*t*) is normalized by the time of one cardiac cycle (T). The shaded area represents the time periods during which positive flow is accompanied by a negative pressure drop, or the reverse flow is accompanied by a positive pressure drop.

The pressure drop between the UA inlet and outlet for the three states during a whole cycle are shown in Figure 11. The Normal pressure drop is totally positive with two peaks and two through values. The LDBF pressure drop shows one positive and one negative peak in a cardiac cycle and remains zero for a period of about 0.42 of a cycle when there is no blood flow. The RDBF pressure drop shows one positive and one negative peak at systole and diastole, respectively, and the UA reverse flow lasts for a period of about 0.4 of a cycle. Generally, the pressure should decrease in the blood flow direction; that is, when the blood flows from the UA inlet to the outlet (positive flow), the pressure drop from the inlet to the outlet also should be positive. However, two interesting findings can be seen in Figure 11. One is that velocity always changes hysteretically after the pressure drop, and the other is that at some certain times in the LDBF and RDBF cases, the positive flow is accompanied by a negative pressure drop, or the reverse flow is accompanied by a positive pressure drop. Actually, the asynchronization of the velocity and the pressure drop is caused by the unsteady nature of the UA blood flow, but this was not considered in previous studies. It is defined as the “hysteresis effect” in this paper and will be discussed further in the following Section 4.

### 3.3. Wall Shear Stress

**Figure 12:**
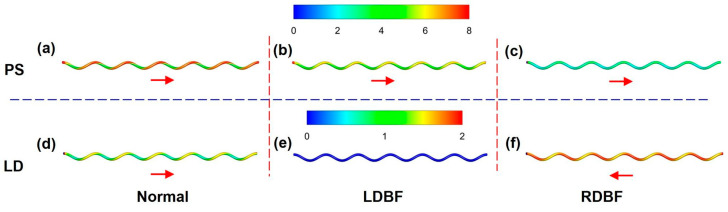
The WSS distribution on the UA wall at the peak systolic (PS (**a**–**c**)) and least diastolic (LD (**d**–**f**)) moments for Normal (**a**,**d**), LDBF (**b**,**e**), and RDBF (**c**,**f**) (in Pa). The arrows represent the actual direction of flow.

**Figure 13:**
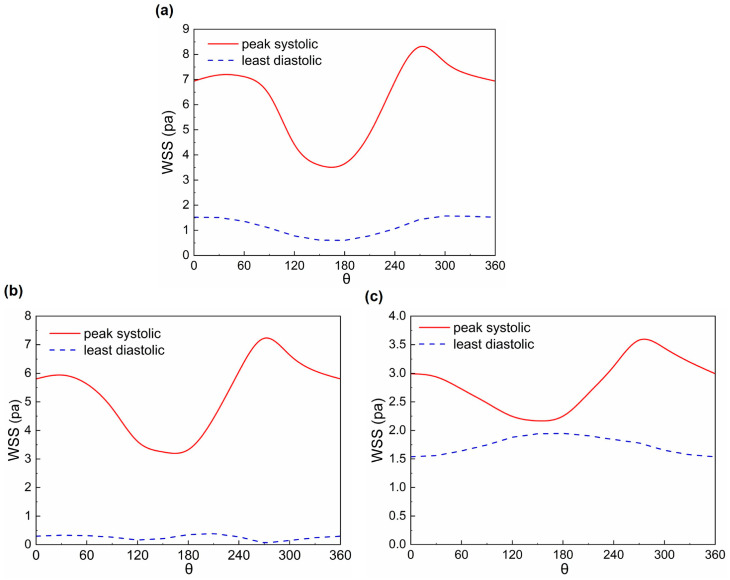
The WSS circumferential distribution in the middle cross-sections of the UA at the peak systolic and least diastolic moments: (**a**) Normal; (**b**) LDBF; and (**c**) RDBF.

Figure 12 shows the WSS distribution of the UA for the three states at the PS and LD moments. The peak WSS at systole is larger than that at diastole for all three states. In addition, the high WSS region for the three states has the same trend at systole and differs at diastole. Figure 13 shows the circumferential distribution of the WSS in the middle cross-section of the UA for the three states, the circumferential angle of which is demonstrated in Figure 2. The systolic circumferential WSS is greater than the diastolic circumferential WSS for all three states. At systole, the minima are all found near 150° on the UA cross-section and the maxima are found near 270°. In contrast, at diastole, the Normal WSS still remains at a minimum value in the cross-section at the 150° position; it was noticed that the RDBF WSS at the 150° position is the maximum, while the LDBF WSS changes very little in the cross-section due to no flow. As can be seen in Figure 10 and Figure 11, at diastole, the RDBF WSS is greater than the Normal WSS.

## 4. Discussion

In this study, Doppler ultrasonic images are used to reconstruct the UA model for CFD simulations. CFD simulations are performed to investigate the hemodynamic characteristics, including the velocity, pressure, and WSS, from normal blood flow (Normal), to the loss of diastolic blood flow (LDBF), and to the reversal of diastolic blood flow (RDBF).

The hysteresis effect between the UA blood flow and the pressure drop is found. As in previous studies [33,37,40], steady UA blood flow is simulated, in which the velocity and pressure drop must be synchronous; that is, the positive flow is associated with a positive pressure drop. Currently, the real periodic pulsatility of UA blood flow based on ultrasound observation is considered, and the blood flow is solved unsteadily. According to Equation (4), in addition to the spatial inertia term $\rho \left(\vec{u}\cdot \nabla \right)\vec{u}$ and viscous term $\nabla \cdot \vec{\tau }$ synchronous with the pressure change term ∇p in time, there is also the unsteady inertia term $\rho \left(\partial \vec{u}/\partial t\right)$ affecting the pressure change, which results in the hysteresis effect. This effect is similar to the braking phenomenon, where although the braking force (as the negative pressure drop) has been exerted, the car still moves on (as the positive velocity).

In the three states, the peak WSS at systole is larger than that at diastole, and the high WSS region has the same trend at systole but is different at diastole. Blood flow friction on the vessel wall generates WSS depending on the vessel diameter, flow rate, and blood viscosity [49]. Saw’s study [37] shows that WSS is not related to the vessel size of the UA, and Shah [33] suggests that the extent of WSS is not affected by changes in the *UCI* and umbilical cord length. The geometric models of the UA in the three states in the present study are consistent. Therefore, it is believed that the differences in the WSS are caused by differences in flow velocities at the entrance of the UA.

One important finding from our study is that the CFD method can explicitly and quantitatively show the normal and IUGR blood flow structure in the UA, while other observation methods can hardly do so. For example, in clinical practice, the Doppler ultrasound mainly displays the estimated direction and velocity of blood flow according to the angle between the ultrasonic beam and the blood flow [50,51]. The hemodynamic information provided by these medical imaging techniques is still limited and not easily readable. CFD shows the streamlines of blood flow, the velocity and instantaneous motion direction of each particle in the fluid, and the flow rate per unit time through a certain section well. CFD can also visually display the pressure and WSS distribution over the UA [52]. Taking the blood flow in the UA as an example, there is a secondary flow on the annular cross-section, which is mixed with the main flow along the direction of the vessel. This kind of movement is difficult to show by ultrasound technology, but the doctor can directly recognize this phenomenon using the present CFD technology. This will be helpful for doctors to better understand the relationship between the UA blood flow and fetal growth restriction.

The second finding is that through the comparison of the structure of the spiral secondary flow, it is found that the distribution of secondary flow patterns differs in the three states’ diastolic period. Firstly, at systole/PS, the direction and the size of the pair of vortices are similar among the three states. Secondly, at diastole, the direction is the same in the normal state as at systole. In LDBF, no blood flows at diastole. The direction is opposite in RDBF between systole and diastole. Thirdly, the size of the pair of vortices and the off-center situation of the velocity are different at systole and diastole in the three states. The three states can represent the successive progression of IUGR in one fetus. Based on this premise, we can regard the difference in the secondary flow as an indicator characterizing the evolution of the condition. The observation of secondary flow can be direct and faster. It can be used in conjunction with other indicators for clinical monitoring in a mutually supportive manner.

The final, new, finding is the hysteresis effect between the blood flow velocity and the pressure drop. To understand IUGR pathologically, placental lesions for the most part lead to damage to the placental structure and function, and the pressure of the UA terminal circulation is simultaneously increased. It is considered that hysteresis can be regarded as a manifestation of the high circulating pressure in the placenta. In LDBF, the reverse conduction of the pressure drop occurs once, and in RDBF, the reverse conduction occurs twice. Therefore, the deterioration of IUGR can be characterized according to the occurrence and frequency of the hysteresis effect.

There are some limitations in this study. Firstly, the elasticity of the UA is not considered, and the UA is considered to be a rigid vessel with a uniform circular cross-section. Secondly, the cells in the blood are not considered, and the blood is considered to be a pure homogeneous liquid phase but with a non-Newtonian property. Thirdly, the spiral state of the UA is different in different fetuses, and the spiral state of the umbilical cord is also different in the same fetus at the same time. This study assumes that the spiral state of the umbilical cord is consistent. With the further development of this research, these factors will be gradually considered to make our research closer to the real situation.

In conclusion, this study uses a UA model to explore the hemodynamic characteristics of the three typical development states of IUGR. From the perspective of fluid dynamics, the CFD results of the UA blood flow allow us to clearly observe the changes in velocity, pressure, and WSS, which has not been explored in depth in the process of disease occurrence and development. More indicators are explored and are promising in the clinical monitoring of IUGR, which may lay the foundation for more accurate monitoring and treatment.

## Figures and Tables

**Table 1:** Grid independent analysis.

Total Number of Grids		Pressure Drop (Pa)	Difference (%)	WSS_avg_ (Pa)	Difference (%)
Mesh 1	440,000	3403.38	1.29	9.35	1.28
Mesh 2	790,000	3359.35	0.05	9.23	0.11
Mesh 3	1,420,000	3357.62	-	9.22	-

**Table 2:** The diameters of the small and large vortices and the ratio of the diameters of the small and large vortices in the middle cross-sections of the UA at the PS and LD moments (*D* is the cross-sectional diameter of the UA).

	Normal			LDBF			RDBF		
	Small	Large	Ratio	Small	Large	Ratio	Small	Large	Ratio
PS	0.2*D*	0.6*D*	0.3	0.2*D*	0.6*D*	0.3	0.4*D*	0.5*D*	0.8
LD	0.3*D*	0.6*D*	0.5	-	-	-	0.2*D*	0.6*D*	0.3

## Data Availability

The data that support the findings of this study are available from the corresponding author upon reasonable request.

## Nomenclature

IUGR	intrauterine growth restriction
UA	umbilical artery
Normal	normal blood flow
LDBF	loss of end diastolic blood flow
RDBF	reversal of end diastolic blood flow
CFD	computational fluid dynamics
WSS	wall shear stress
PS	peak systolic
LD	least diastolic
UC	umbilical cord
UV	umbilical vein
*UCI*	umbilical cord index
3D	three-dimensional
*R_r_*	spiral radius of the UA, m
*R_A_*	radius of the UA, m
*R_V_*	radius of the UV, m
*U*	number of UA spirals
*L*	length of the UC, cm
*P_h_*	UA pitch, cm
*x*, *y*, *z*	Cartesian coordinate system coordinate variables
$\vec{u}$	fluid velocity vector, m/s
*p*	pressure, Pa
*ρ*	blood density, kg/m^3^
$\vec{\tau }$	viscous stress tensor, Pa
*μ*	blood viscosity, pa·s
*γ*	local shear rate, s^−1^
*λ*	time constant, s
*n*	power exponent
*μ*_0_	zero shear viscosity, Pa·s
*μ_∞_*	infinite shear viscosity, Pa·s
Re	Reynolds number
Re_m_	maximum of Reynolds number
*R*	tube radius, m
*D*	diameter of UA, m
*U_m_*	maximum average speed of blood flow, m/s
*μ* *_a_*	average dynamic viscosity of blood, Pa·s
T	cardiac cycle, s
Δ*t_i_*	time marching step for simulation, s, i = 1, 2, 3
Dn	Dean number
k	normalized curvature
*b*	parameter indicating pitch, cm
*t*	time, s
